# Primary silicone oil tamponade and internal limiting membrane peeling for retinal detachment due to macular hole in highly myopic eyes with chorioretinal atrophy

**DOI:** 10.1186/s12886-015-0154-4

**Published:** 2015-11-11

**Authors:** Nan Hong, Bai-shuang Huang, Jian-ping Tong

**Affiliations:** Department of Ophthalmology, the First Affiliated Hospital of College of Medicine, Zhejiang University, Hangzhou, Zhejiang P. R. China

**Keywords:** Myopia, Macular hole, Retinal detachment, Chorioretinal atrophy

## Abstract

**Background:**

Retinal detachment (RD) secondary to macular hole (MH) is a common complication in highly myopic eyes, usually leading to a poor visual prognosis. The purpose of this study was to evaluate the surgical outcome of silicone oil (SO) tamponade and internal limiting membrane (ILM) peeling in the treatment of RD caused by MH (MHRD) in highly myopic eyes with chorioretinal atrophy, and to identify clinical factors associated with the anatomical outcomes.

**Methods:**

We retrospectively reviewed 21 eyes of 21 highly myopic patients affected by RD secondary to MH and chorioretinal atrophy. All eyes were treated with pars plana vitrectomy (PPV) with ILM peeling and SO tamponade. Anatomical success was defined as reattachment of the retina with the closure of the MH, as assessed by optical coherence tomography (OCT), after SO removal. Logistic regression was performed to determine the clinical factors influencing anatomical success.

**Results:**

The mean patient age was 59.95 years [standard deviation (SD), 10.39; range, 34–77 years] and the mean axial length was 30.58 mm (SD, 1.52; range, 27.99–34.51 mm). After the first surgical procedure, the anatomical success rate was 61.9 % (13 eyes in 21 eyes), with initial retinal attachment of16 eyes (76.2 %). A second surgical approach was performed for the five eyes with persistent or recurrent RD, and the final retinal reattachment rate was 100 % (21/21). Logistic regression analysis showed that no specific factors were significantly associated with anatomical success.

**Conclusions:**

Primary silicone oil tamponade and ILM peeling can be a practical treatment for repairing MHRD in highly myopic eyes with chorioretinal atrophy.

## Background

Retinal detachment (RD) secondary to macular hole (MH) is a common complication in highly myopic eyes, usually leading to a poor visual prognosis. The precise pathogenic mechanism underlying the condition remains unclear. Many factors such as anteroposterior/tangential vitreous traction, posterior staphyloma, and chorioretinal atrophy have been proposed as contributing to its onset [1, 2]. In addition, vascular stiffness and internal limiting membrane (ILM) have also been reported as tractional forces that may lead to retinal detachment secondary to macular hole (MHRD) in highly myopic eyes [3].

Many surgical approaches have been used to repair MHRD in highly myopic eyes, including pneumoretinopexy, pars plana vitrectomy (PPV) with gas, silicone oil (SO) or heavy SO tamponade, and macular buckling (MB) [2, 4]. Tangential traction exerted by the epiretinal membrane (ERM) has been reported to be a major causative factor for MHRD. To peel the ERM around the MH would increase the flexibility of the retina, contributing to the closure of the MH [4–7]. Peeling the ILM has also been performed as a surgical adjunct to facilitate closure of the MH. Myofibroblasts on the inner surface of the ILM contact tissues of the retina around the macular; therefore, peeling the ILM would remove the scaffold for cellular proliferation, releasing any tractional components affecting the retina. Moreover, removal of the ILM can result in complete removal of the ERM, leading to MH sealing [6]. However, it is unquestionable that MB still remain the only available technique which allows to physically counteract the anteroposterior traction exerted by the staphyloma [8].

In highly myopic eyes with chorioretinal atrophy, surgical treatment for RD resulting from MH is difficult. Based on funduscopic findings, the myopic chorioretinal atrophies are classified into diffuse chorioretinal atrophy (DCA) or patchy chorioretinal atrophy (PCA) [9]. Diffuse chorioretinal atrophy is defined as yellowish-white lesions without clear borders, with the visual acuity not severely affected. Patchy chorioretinal atrophy is defined as a grayish-white and well-defined lesion, characterized by a complete loss of the choriocapillaris, leading to an absolute scotoma in the foveal area. In the present study, we retrospectively reviewed highly myopic MHRD patients with PCA around the MH who had been treated with PPV, ILM peeling, and primary silicone oil tamponade. We also investigated the prognostic factors associated with anatomical success in these patients.

## Methods

This retrospective study was approved by the Institutional Review Board of the First Affiliated Hospital, Medical College of Zhejiang University, and adhered to the tenets of the Declaration of Helsinki. Each of the patients was informed about the nature of the study, and written informed consent was obtained. We reviewed the medical records of 21 consecutive eyes of 21 myopic patients with RD due to MH. All patients underwent PPV with indocyanine green (ICG)-assisted ILM peeling surgery and primary silicone oil tamponade. The surgeries were performed by a single surgeon (J.P.T.) between February 2009 and July 2015 at the First Affiliated Hospital, Medical College of Zhejiang University. The inclusion criteria were: (1) patients with clinical presentation of RD caused by a MH in a myopic eye [refractive error >−6.00 diopters (D) and axial length (AXL) > 26.5 mm]; (2) treatment with a 23-gauge 3-port PPV with the ILM peeling and primary silicone oil tamponade; (3) a postoperative follow-up period more than 6 months after the primary vitrectomy; and (4) the existence of PCA on the background of the MH in the surgical eye. The exclusion criteria included: (1) previous ocular surgery, except for phacoemulsification; (2) a traumatic MH; and (3) a history of other ocular disease or systemic disease such as diabetes mellitus.

All patients underwent comprehensive ophthalmic examinations before and after the surgery, including best-corrected visual acuity (BCVA), slit-lamp biomicroscopy, fundus examination, intraocular pressure (IOP) determination, time-domain optical coherence tomography (TD-OCT), B-scan ultrasonography, and IOL master. AXL was measured by B-scan ultrasonography. All color fundus images were obtained in 1 month after the first surgery. The area of the PCA was defined according to the following findings in fundus features: (1) a PCA within the vascular arcade or restricted to the posterior pole or (2) a PCA also extending beyond the vascular arcades. Information of each case included age, sex, pre- and postoperative BCVA, the duration of RD, AXL, preoperative lens status, area of the PCA, the duration of silicone oil tamponade, MH closure, and retinal reattachment and complications. The presence of posterior staphyloma in all patients was also noted according to the fundus examination and B-scan ultrasonography.

A standard 23-gauge pars plana vitrectomy was performed under retrobulbar anesthesia on all patients, followed by the dissection of the posterior hyaloid with triamcinolone acetonide staining. If present, the ERM was removed. Afterward, ILM peeling assisted by ICG staining was performed on all patients. During the procedure, perfluorocarbon was not used. In addition, a vitreous base shaving or a 360 laser photocoagulation was not performed. Fluid-air exchange and tamponade with silicone oil (5,000 centistokes) were used at the end of the surgical procedures. After surgery, patients were instructed to maintain a prone position for at least 7 days. A combined cataract surgery was performed when the obscured lens influenced the PPV procedures.

All patients were followed weekly in the first month after the first surgery, and every 3 months thereafter. After the initial surgery, the surgical technique of SO removal was performed for patients whose retina was reattached, as assessed by OCT, for at least 3 months, regardless of whether the MH was opened or closed. The closure of MH was defined by the presence of MH edge re-approximation [10]. Anatomical success was defined as re-attachment of the retina with the closure of the MH, as assessed by OCT, after silicone oil removal.

### Statistical analysis

The BCVA was converted to the logarithm of the minimal angle of resolution (logMAR) for statistical analyses. Visual acuity of counting fingers and hand motions was arbitrarily assigned an equivalent of 2.3 and 2.6 logMAR units, respectively. Statistical analyses were performed with SPSS, version 18.0 (SPSS Inc., Chicago, IL, USA). The preoperative and postoperative BCVAs were compared using a paired-sample *t* test. For continuous variables, the Mann–Whitney *U* test was performed to analyze their differences. The chi square test or Fisher’s exact test was used for categorical variables. Logistic regression models were used to assess the independent effects of specific preoperative clinical factors on anatomical success rates. The clinical factors included age, AXL, area of the PCA, combined phacoemulsification with IOL implantation, duration of silicone oil tamponade, and duration of the RD. A value of *P* < 0.05 was considered statistically significant.

## Results

In our study, a total of 21 eyes of 21 patients were included (two male and 19 female patients). The mean patient age was 59.95 years [standard deviation (SD), 10.39 years; range, 34–77 years]. The mean AXL was 30.58 mm (SD, 1.52; range, 27.99–34.51 mm). All patients were followed up for 24 months. Combined cataract surgery was performed in 12 eyes (57.1 %). Nine patients did not receive cataract surgery; four patients were pseudophakic and the other five patients still had clear lenses. Until the removal of SO, the lenses of the five patients were not obscured, so we did not perform cataract surgery. All the patients had posterior staphyloma. Demographic data are shown in Table 1.

**Table 1:** Clinical characteristics of patients with MHRD who received PPV with silicone oil tamponade and ILM peeling

No.	Sex	Age	AXL	Pre BCVA	Post BCVA	Area of PCA	Cataract surgery	Duration of RD (ms)	Duration of SO (ms)	Duration of follow-up (ms)	Initial MH closure	Initial Retinal attachment
1	F	69	29.45	0.02	FC	1	1	6	5	24	NO	NO
2	F	71	30.13	HM	0.06	0	0	0.6	5	24	YES	YES
3	F	54	31.29	0.02	0.06	1	0	0.5	5	24	YES	YES
4	F	77	30.68	FC	0.1	0	1	2	9	24	YES	YES
5	F	46	28.76	0.04	0.08	1	1	0.3	9	24	YES	YES
6	F	62	30.44	FC	0.12	0	0	0.5	12	24	YES	YES
7	F	64	31.30	0.02	0.04	0	1	2	4	24	YES	YES
8	F	61	27.99	HM	0.01	1	1	1	8	24	NO	NO
9	F	62	30.67	0.02	0.02	1	1	1	10	24	YES	YES
10	F	63	29.54	0.01	0.04	1	1	0.6	7	24	NO	NO
11	F	34	28.51	FC	0.05	0	0	0.1	6	24	YES	YES
12	F	66	33.20	FC	0.02	0	1	0.5	12	24	YES	YES
13	F	57	30.13	FC	0.02	1	0	4	12	24	NO	NO
14	F	71	29.25	HM	0.05	0	1	0.6	18	24	YES	YES
15	F	62	31.82	0.06	0.1	1	0	0.2	6	24	NO	YES
16	F	58	30.38	HM/30	0.04	1	0	0.3	5	24	NO	YES
17	F	65	30.95	HM	FC	1	0	0.3	4	24	YES	YES
18	F	54	31.09	FC	0.02	1	1	0.6	15	24	YES	YES
19	F	70	32.02	FC	0.06	0	1	0.6	12	24	YES	YES
20	M	50	30.14	0.02	0.01	1	1	0.3	5	24	NO	NO
21	M	43	34.51	0.12	0.02	1	0	0.6	15	24	NO	YES

*AXL* axial length, *BCVA* best corrected visual acuity, *PCA* patchy chorioretinal atrophy, *RD* retinal detachment, *SO* silicone oil, *MH* macular hole, *HM* hand motion, *FC* finger counting, *F* female, *M* male. Area of PCA: 1 = Within the vascular arcade; 0 = Beyond the vascular arcade. Cataract surgery: 1 = YES; 0 = NO

After the first surgical procedure, the anatomical success rate (defined as both MH closure and retinal reattachment) was 61.9 % (13 eyes in 21 eyes), 16 eyes (76.2 %) had initial retinal attachment with or without MH closure. Because some patients did not follow up on time, the SO removal surgery could not be performed within 6 months of the primary surgery. The mean period of silicone oil tamponade was 8.95 months (SD, 4.27 range, range, 4–18 months), and all patients had silicone oil removal (Table 2).

**Table 2:** Baseline clinical factors of highly myopic patients with MHRD

Clinical Factors	All cases (*n* = 21)
Mean age ± SD, years (range)	59.95 ± 10.39 (34 to 77)
Gender	
Male	2
Female	19
Mean AX ± SD, mm (range)	30.58 ± 1.52 (27.99 to 34.51)
Duration of RD ± SD, months (range)	1.07 ± 1.43 (0.1 to 6)
Duration of SO tamponade ± SD, months (range)	8.95 ± 4.38 (4 to 18)
Duration of Follow-up ± SD, months (range)	24.29 ± 13.95 (10 to 69)
Area of PCA, n (%)	
Within the vascular arcade	11 (52.4 %)
Beyond the vascular arcade	10 (47.6 %)
Combined cataract surgery, n (%)	
Yes	12 (57.1 %)
No	9 (42.9 %)

**Fig. 1:**
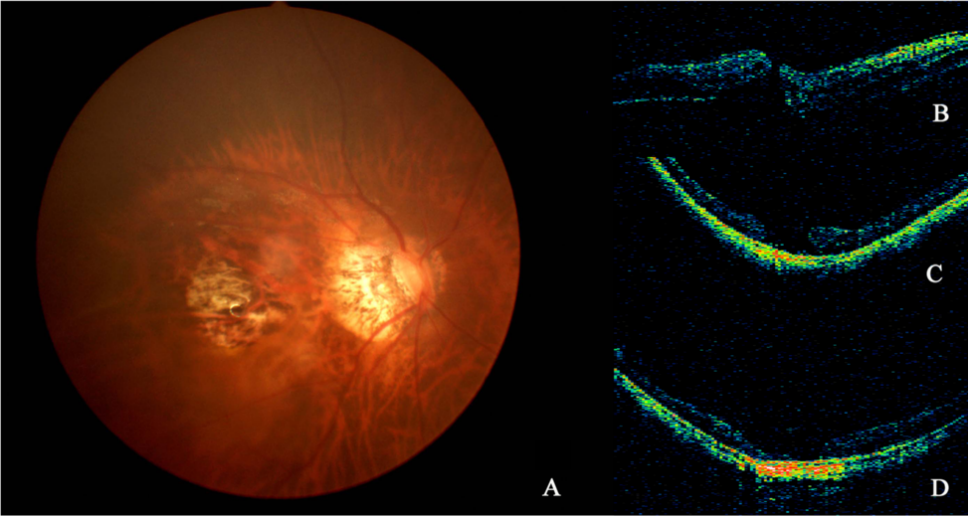
Fundus photograph and OCT images (**a**, **b**, **c** and **d**) from Patient 1. **a** PCA around MH was shown on fundus photograph. **b** RD caused by MH developed. **c** After the SO removal, retinal reattachment was achieved, MH was still open on OCT. **d** After the second silicone oil removal, the patient achieved retina reattachment despite persistent open of the MH

**Fig. 2:**
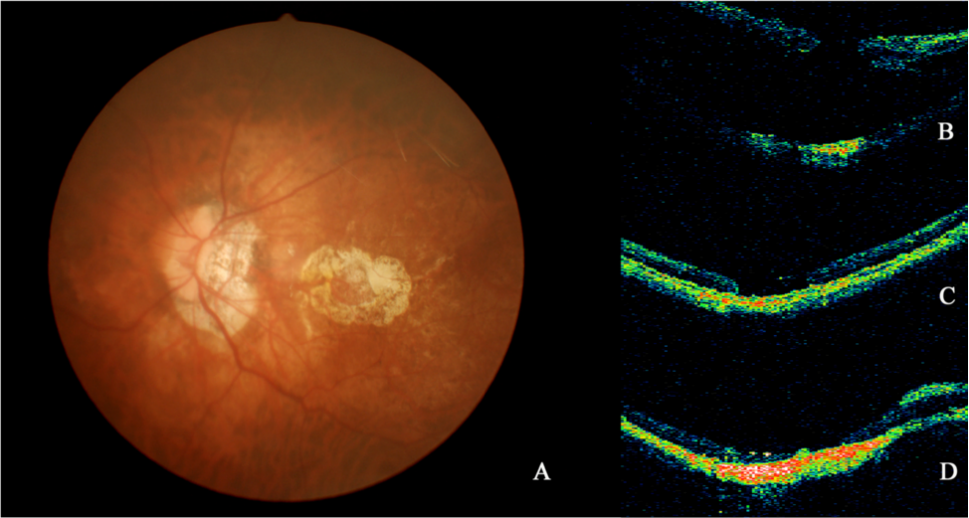
Fundus photograph and OCT images (**a**, **b**, **c** and **d**) from Patient 20. **a** PCA around MH was shown on fundus photograph. **b** RD caused by MH developed. **c** After the SO removal, retinal reattachment was achieved, MH was still open on OCT. **d** After the second silicone oil removal, the patient achieved retina reattachment despite persistent open of the MH

Eight out of 21 patients (39.1 %) had a persistent MH and in five of them, the retina was still detached (23.8 %). Among these five cases without initial retinal reattachment, three had a persistent RD under SO tamponade, while the other two had a recurrent RD 2 weeks and 1 month after SO removal, respectively. The cause of recurrent RD mainly included reopening of the MH and recurrence of ERM. For the three eyes with a persistent RD, a second surgical approach with SO removal and perfluoropropane (C3F8) tamponade was performed. In addition, peeling of recurrent ERM was performed. The other two patients with recurrent RD after SO removal underwent a second surgery with further SO tamponade (5,000 centistokes). Both of them achieved a final retinal reattachment despite a persistent MH after final SO removal (Figs. 1 and 2). The final MH closure rate was 76.2 % (16/21), and the final retinal reattachment rate was 100 % (21/21). The relationships between clinical factors and anatomical successes are listed in Table 3. Logistic regression analysis showed that no specific factors were significantly associated with anatomical successes (Table 4).

**Table 3:** Relationship between anatomical success and clinical factors in highly myopic eyes with MHRD

Clinical factors	Anatomical success		*P* value
	Yes (n = 13)	No (n = 8)	
Mean age, years (range)	61.23 (34–77)	57.88 (43–69)	0.217^a^
Mean AL, mm (range)	30.64 (28.51–33.2)	30.5 (27.99–34.51)	0.447^a^
Mean duration of RD, ms (range)	0.74 (0.1–2)	1.63 (0.2–6)	0.685^a^
Mean duration of SO tamponade, ms (range)	9.62 (4–18)	7.88 (5–15)	0.511^a^
Area of PCA, n			0.024^b^
Within the vascular arcade	4	7	
Beyond the vascular arcade	9	1	
Combined with cataract surgery, n			0.673^b^
Yes	8	4	
No	5	4	

^a^Mann–Whitney *U* test

^b^Fisher’s exact test

**Table 4:** Logistic regression analysis of clinical factors for anatomical success in highly myopic eyes with MHRD

Clinical factor	Odds ratio (95 % CI)	*P* value
Age (y),	1.023 (0.893–1.172)	0.740
AL (mm),	1.128 (0.421–3.025)	0.810
Duration of RD	0.421 (0.057–3.127)	0.398
Duration of SO tamponade	1.056 (0.739–1.508)	0.765
Area of PCA	0.064 (0.003–1.198)	0.066
Combined with cataract surgery	1.169 (0.064–21.366)	0.916

Postoperative BCVA improved in 16 eyes (76.2 %), was unchanged in two eyes (9.5 %), and worsened in three eyes (14.3 %). The initial mean BCVA (logMAR) was 2.07 (SD, 0.45, range, 1.17–2.6), and increased to 1.51 (SD, 0.44, range, 0.9–2.3) at the end of the follow-up. Although there was a significant difference in the BCVA before and after surgery (*P* = 0.000, t = 4.168, 95 % confidence intervals 0.28–0.84, paired *t* test), only one eye had a postoperative BCVA > 0.1.

Ten eyes (45.5 %) had a high IOP (>21 mmHg) from the first postoperative day, and nine eyes returned to normal IOP levels within 2 weeks using anti-glaucomatous eye drops. In one eye (case 21) with a high IOP (> 35 mmHg) that could not be controlled with anti-glaucomatous eye drops for days, Ahmed glaucoma valve implantation was performed 2 weeks after the silicone oil tamponade, then treated with anti-glaucomatous eye drops until oil removal. During the silicone oil tamponade, the IOP of the patient was normal. After the removal of SO, none of the patients had a high IOP. No peripheral rhegmatogenous RD was reported during the follow-up period. Silicon oil emulsification was not observed in the anterior chambers or vitreous chambers in all patients. Other intraocular complications such as severe intraocular inflammation were not observed.

## Discussion

The preferred surgical treatment for RD caused by a MH remains controversial. The application of PPV combined with gas tamponade and ILM peeling have been used to repair MHRD in eyes with a MH [11, 12]. In cases of RD caused by a MH in myopic eyes with marked chorioretinal atrophy and posterior staphyloma, the sole use of gas tamponade is insufficient. The loss of the choriocapillaris leads to poor retinal adhesion to the underlying pigment epithelium, and retinal adhesion could be overcome by the posterior staphyloma that produces an inverse traction [13]. More aggressive surgical procedures such as silicone oil tamponade or PPV combined with MB might therefore be needed [14]. In the present study, we performed PPV with silicone oil tamponade and ILM peeling to treat RD ressoulting from a MH in highly myopic eyes with chorioretinal atrophy, achieving an initial retinal reattachment rate of 76.2 %.

Recent studies on RD caused by a MH treated by PPV plus primary silicone oil tamponade with ILM peeling have suggested a high reattachment rate ranging from 85.1 to 100 % [15–18]. Nishimura et al. reported that PPV with ILM peeling and primary silicone oil tamponade without any postoperative position restrictions led to a 92 % retinal reattachment rate after the initial surgery, with a mean duration of silicone oil tamponade of 23.3 months [17]. In another study by Meng et al. [19] in 21 consecutive highly myopic eyes with RD resulting from MH, the MH closure rate was 86 % and the final retinal reattachment rate was 95.2 %. However, the occurrence of chorioretinal atrophy in patients was not reported in these studies. Nadal et al. [20] studied a series of 27 highly myopic eyes that presented with posterior pole RD and MH treated with PPV combined with silicone oil tamponade and ILM dissection to the edge of the posterior staphyloma, with a retinal reattachment rate of 85.1 %. However, the degree of chorioretinal atrophy was not described in their study. In the present study, the initial retinal reattachment rate was 76.2 %. This was lower than previously reported, because the cases included those with patchy chorioretinal atrophy around the MH and posterior staphyloma, representing a more serious condition compared with previous reports.

Clinical risk factors associated with anatomical success and MH closures in highly myopic eyes were difficult to determine. In a retrospective study by Nishimura et al. [17], six clinical factors (age, preoperative BCVA, AXL, area of the RD, combined cataract surgery, and the duration of oil tamponade) were assessed, and all these factors were not correlated with MH closure. Xie et al. [18] assessed clinical risk factors associated with initial anatomical success of MHRD treated with primary silicone oil tamponade with or without ILM peeling, and found that MHRD in myopic eyes with smaller MH diameters were significantly associated with higher initial anatomical successes. The most recent study by Arias et al. reported that the area of the fundus autofluorescence and AXL were significantly associated with anatomical outcomes [20]. In the present study, we did not include refraction because it might be affected by the intraocular lens or silicone oil. Other potential clinical prognosis factors such as MH diameters [21], location of RD, the presence of proliferative vitreous retinopathy (PVR), and posterior vitreous detachment (PVD) were not included because information of the cases was incomplete. According to Curtin, the posterior staphyloma in highly myopic eyes can be classified into 10 types. Types I–V are primary staphyloma, and types VI–X are combined staphyloma [22]. However, this classification may not be reliable and objective, because it was only derived from ophthalmoscopic appearances and fundus drawings [23]. Hence, the type of posterior staphyloma was not included in the present study. Eventually, six clinical factors were analyzed in our study, including age, AXL, area of the PCA, the duration of the MHRD, the duration of the SO tamponade, and the combination of cataract surgery. Regarding the variables in the logistical regression model, no specific clinical factors were significantly correlated with anatomical outcomes. This result was contradictory to previous reports, mainly owing to the small sample size of our study.

For eyes with persistent RD, treatment options are challenging. Nishimura et al. [17] reported two eyes with recurrent RD that were treated by MB, both resulting in final retinal reattachment. Xie et al. [18] reported six eyes that experienced recurrences of RD before silicone oil removal. In their study, three of the eyes achieved final retinal reattachment by the patient remaining in a strict prone position for at least 1 week after silicone removal. Two eyes had subretinal fluid drained by placing a flute needle under the surface of the silicone oil without peeling the membrane. Both of them achieved final retinal reattachment; the other eye also achieved final retinal reattachment after silicone oil removal with ERM peeling and gas tamponade. In the present study, we performed silicone oil removal and gas tamponade combined with recurrent ERM peeling for three eyes with recurrent RD before silicone removal. All eyes achieved final retinal reattachment. Any of these surgical procedures could be chosen to treat eyes with recurrent RD, although the choice of optimal surgery needs further prospective studies.

Regarding the study by Xie et al.[18], maintaining a strict prone position postoperatively might provide sufficient compressive force to the macular area, because the surface tension of silicone oil was not sufficient to counteract the stretching force around the MH in myopic eyes, especially in eyes with chorioretinal atrophy. In the present study, we also advised patients to keep a strict prone position postoperatively.

In our study, the postoperative BCVA of 20 patients (95.2 %) was < 0.1. The presence of patchy chorioretinal atrophy and decreased retinal sensitivity limited the recovery of functional outcomes. Furthermore, all eyes in the study had a thin retina and choroid, making them more vulnerable to the toxicity of the ICG dye, leading to a poor visual outcome, probably owing to both retinal and optic nerve damage related to the use of silicone oil [24].

The disadvantages of silicone oil tamponade include elevation of IOP and the need of a second surgical procedure. In the present study, one patient had a high IOP > 35 mmHg after the initial surgery that could not be controlled by anti-glaucomatous eye drops for weeks. Ahmed glaucoma valve implantation was therefore performed 2 weeks after silicone oil tamponade. Though the patient achieved final retinal reattachment, the visual acuity decreased postoperatively.

The limitations of the present study include a small sample size, a retrospective design, a lack of a controlled study group, and a short follow-up time (24 months). Further prospective cohort studies should therefore be performed to confirm our conclusions.

## Conclusions

PPV with peeling of ILM and silicone oil tamponade can be an effective treatment for repairing MHRD in highly myopic eyes with chorioretinal atrophy. A postoperative prone position might be helpful for anatomical recovery.

## Abbreviations

AXL: Axial length
BCVA: Best-corrected visual acuity
DCA: Diffuse chorioretinal atrophy
ERM: Epiretinal membrane
ICG: Indocyanine green
ILM: Internal limiting membrane
IOP: Intraocular pressure
MB: Macular buckling
MH: Macular hole
OCT: Optical coherent tomography
PCA: Patchy chorioretinal atrophy
PPV: Pars plana vitrectomy
PVD: Posterior vitreous detachment
PVR: Proliferative vitreous retinopathy
RD: Retinal detachment
RPE: Retinal pigment epithelium
SD: Standard deviation
SO: Silicone oil
